# Superspreading-Based Fabrication of Poly(methyl methacrylate) Films with High Toughness for Ultra-Wideband Flexible Transparent Antenna

**DOI:** 10.3390/ma18102183

**Published:** 2025-05-09

**Authors:** Ying Liu, Cheng Huang, Jiannan Guo, Haoran Zu, Jie Shen, Pengchao Zhang, Wen Chen

**Affiliations:** 1 Hubei Longzhong Laboratory, Wuhan University of Technology Xiangyang Demonstration Zone, Xiangyang 441000, China; 331029@whut.edu.cn (Y.L.); chenw@whut.edu.cn (W.C.); 2 Key Laboratory of Advanced Technology for Materials Synthesis and Processing, School of Materials Science and Engineering, Wuhan University of Technology, Wuhan 430070, China; klhuangcheng@hotmail.com; 3 Hubei Engineering Research Center of RF-Microwave Technology and Application, School of Physics and Mechanics, Wuhan University of Technology, Wuhan 430070, China; gjn0419@whut.edu.cn; 4 School of Information Engineering, Wuhan University of Technology, Wuhan 430070, China; zuhr@whut.edu.cn; 5 Sanya Science and Education Innovation Park, Wuhan University of Technology, Sanya 572024, China

**Keywords:** superspreading, PMMA films, high toughness, flexible transparent antenna

## Abstract

With the rapid advancement of Internet of Things (IoT) technology, ultra-wideband flexible transparent antennas have garnered substantial attention for their potential applications in wireless communication devices. Poly(methyl methacrylate) (PMMA), renowned for its exceptional optical properties and favorable processing characteristics, has been extensively utilized as a transparent substrate material for antennas. However, the intrinsic brittleness of transparent PMMA substrates poses a significant limitation in applications such as flexible antennas. In this study, we introduce a superspreading strategy to address the complex trade-off among transparency, toughness, and dielectric properties in flexible electronics through molecular disorder engineering. The PMMA films fabricated via this superspreading strategy exhibit a visible transmittance of 85–95% at 400 nm, a toughness of 9 × 10⁵ J/m^3^ (representing an enhancement of 150–225% compared to conventional methods), and a frequency-stable permittivity (ε_r_ = 3.6 ± 0.05) within the 9–12 GHz range. These films also feature a precisely tunable thickness range of 5.5–60 μm. The PMMA-based flexible transparent antenna demonstrates a gain of 2–4 dBi and a relative bandwidth of 40%, thereby confirming its suitability for ultra-wideband applications. Collectively, this research presents a promising candidate for the development of ultra-wideband flexible transparent antennas.

## 1. Introduction

Ultra-wideband flexible transparent antennas have garnered substantial interest, as they provide the technical benefits required for high-performance communication while also offering the aesthetic advantage of concealability [1]. Notably, their strain-sensitive impedance characteristics enable dual functionality as conformal deformation sensors, allowing real-time structural health monitoring through radio frequency signal variations [2,3]. This ability to seamlessly integrate into diverse environments without compromising performance makes them highly suitable for incorporation into Internet of Things (IoT) applications and everyday devices. Among potential candidates for antenna substrates, polymer films exhibit several notable advantages, particularly in terms of frequency selectivity, manufacturing cost, and flexibility [4,5,6]. Various polymer films, including polyimides [7], polyethylene terephthalate [8], and poly(methyl methacrylate) (PMMA), have been explored for the development of flexible transparent antennas. Among these, PMMA has emerged as a promising candidate due to its high transparency and favorable dielectric properties [9,10,11,12,13]. A variety of film-forming methods have been employed to prepare PMMA films, including solution casting, spin coating, nonsolvent-induced phase separation, and flow-delaying techniques [14,15,16,17]. However, these methods face significant challenges in enhancing the overall mechanical performance of the resulting films, particularly in terms of toughness. While solution casting offers operational simplicity, the resultant films often exhibit poor mechanical integrity, characterized by excessive brittleness [18,19]. Spin coating, although enabling rapid fabrication, frequently leads to structural heterogeneity within the film matrix due to accelerated solvent evaporation, thereby compromising toughness [20,21,22]. Consequently, the low toughness of PMMA films fabricated by existing methods severely limits their applicability in flexible antenna systems that require durable deformation tolerance.

To address this issue, various modification methods have been proposed, including copolymerization, nanoparticle doping, and crosslinking [23,24,25]. For instance, Tong et al. developed PMMA films with enhanced modulus through copolymerization; however, the poor compatibility of the monomers results in localized stress concentration points, which severely limit the improvement of toughness [23]. Iqbal et al. achieved mechanical reinforcement via radiation crosslinking, but the induced molecular chain constraints actually reduce crack propagation resistance—a critical indicator of toughness—alongside compromised optical transparency [24]. Kamonkhantikul et al. demonstrated that a nanoparticle-doped system can increase toughness, but the agglomerated particles create interfacial weak zones within the matrix. These interface layers can lead to uneven local electric field distribution, which in turn increases dielectric loss [25]. While these strategies emphasize conventional mechanical metrics, their optimization of molecular entanglement dynamics within PMMA’s amorphous architecture remains incomplete. These approaches often sacrifice optical transparency and dielectric properties. The disordered polymeric network, which is vital for enabling chain mobility that governs energy dissipation and crack resistance, has not been systematically engineered. Consequently, to advance PMMA for applications requiring both mechanical resilience and electrical precision, innovative molecular designs must reconcile chain mobility for energy dissipation with structural homogeneity for electron confinement.

For amorphous materials, the degree of molecular chain disorder is a critical determinant of mechanical performance of polymer films. The molecular disorder enhances the capacity for energy absorption, thereby improving the toughness and impact resistance of the material [26,27]. To capitalize on this effect, we develop a superspreading strategy [28,29,30]. This methodology strategically exploits the intrinsic molecular disorder of the polymer matrix, where optimized heterogeneity directly enhances mechanical energy dissipation and dielectric uniformity. Such a superspreading strategy has been employed to continuously fabricate thin polymer films, such as polyimide [31], poly(vinylidene fluoride) [32]. Notably, the poly(vinylidene fluoride) (PVDF) films fabricated by the superspreading method have been successfully applied in high-performance thin-film dielectric capacitors [33].

Building upon these fundamental insights, we demonstrate the successful integration of superspreading technology into PMMA thin-film fabrication, establishing a conformational disorder-mediated optimization paradigm. The demonstrated synergy between disordered polymer architectures and multifunctional performance opens avenues for scalable fabrication of flexible electronics capable of seamless integration into conformal antennas.

## 2. Materials and Methods

### 2.1. Materials

Poly(methyl methacrylate) (PMMA) homopolymer (molecular weight, Mw ≈ 180,000) with a refractive index of 1.49 was procured from Aldrich Chemical Company (St. Louis, MO, USA). N,N-Dimethylformamide (DMF) was obtained from Chemical Reagents Limited Company (Waltham, MA, USA). These materials were utilized as received, without undergoing any further modifications.

### 2.2. Sample Preparation

A total of 5 g of PMMA powder was dissolved in DMF at ambient temperature to prepare solutions with concentrations of 4 wt%, 5 wt%, 6 wt%, and 7 wt%. The mixture was subsequently subjected to ultrasonication for 24 h using a magnetic stirrer, followed by an additional 2 h of ultrasonication in an ultrasonic cleaner to ensure complete dissolution and formation of a homogeneous, transparent solution. A coagulation solution was prepared by mixing deionized water with DMF, with the volume ratio of water to DMF controlled between 20:80 and 70:30. The PMMA solution was then slowly poured onto the surface of the coagulation bath, where it spread autonomously, forming a porous PMMA film in the wetted state. The film was subsequently immersed in deionized water for 30 min to facilitate solvent exchange and removal of any residual solvent. The membrane was then air-dried at room temperature for 48 h and subjected to heat treatment at 200 °C for 10 min to obtain a transparent, dense film.

### 2.3. Characterization of PMMA Films

Two-dimensional X-ray scattering analyses were systematically conducted to investigate the microstructural evolution of the polymeric films. Wide-angle X-ray diffraction (2D-WAXD) and small-angle X-ray scattering (2D-SAXS) measurements were performed using a synchrotron-grade X-ray scattering system (Xeuss 2.0, Xenocs SA, Grenoble, France) equipped with a hybrid pixel detector (Eiger2R 1M, Dectris AG, 75 μm pixel resolution, Baden, Switzerland). Monochromatic Cu Kα radiation (λ = 1.54189 Å, 8.05 keV) was generated via a rotating anode source with multilayer optics. The sample-to-detector distances were calibrated to 50 mm for the WAXD configuration and 750 mm for the SAXS configuration, respectively, enabling simultaneous acquisition of crystalline lattice information and nanoscale phase separation features through azimuthally integrated diffraction pattern analysis. Nexus intelligent Fourier-transform infrared (FTIR) spectroscopy was conducted in attenuated total reflectance (ATR) mode, with a scanning wavenumber range of 4000 to 600 cm^−1^. The morphology of the film was investigated using a scanning electron microscope (SEM) (Zeiss Ultra Plus, Carl Zeiss, Oberkochen, Germany) operated at an accelerating voltage of 10 kV. The cross-section of the film was obtained by immersion in liquid nitrogen, followed by sputter-coating with a thin layer of gold to enhance conductivity. The mechanical strength of the films was evaluated using a tension testing machine (Mark-10 series F105, Copiague, NY, USA). UV-vis transmittance spectra (200–800 nm) were acquired using a dual-beam spectrophotometer (UV-2600, Shimadzu Corp., Kyoto, Japan) equipped with an integrating sphere attachment. The dielectric properties of the PMMA films were assessed using a Vector Network Analyzer model (WR-90, Pasternack, Irvine, CA, USA) via the waveguide method in the frequency range of 9–12 GHz. The film samples, sized 22.86 mm × 10.16 mm, were placed between two waveguide coaxial converters with aligned waveguide apertures, and the waveguide coaxial converters were connected to a calibrated vector network analyzer.

### 2.4. Antenna Performance Metrics

Reflection coefficient measurements were conducted using a PNA-X Vector Network Analyzer (VNA) (Keysight N5247A, Roseville, CA, USA) across the 8–12 GHz frequency band. Prior to measurement, a full two-port calibration was performed using the Keysight 85058B metrology-grade calibration kit to eliminate systematic errors, including coaxial cable losses, connector discontinuities, and test equipment imperfections. This calibration procedure established precise reference planes at the antenna feed points. For radiation pattern characterization and gain quantification, measurements were performed in an anechoic chamber compliant with IEEE 1720-2012 standards [34] for far-field conditions. The three-antenna gain derivation method was employed: First, the transmission coefficient (S_21_) between two reference horns was recorded to establish the baseline system loss. Subsequently, the device under test (DUT) replaced one reference horn for differential S_21_ measurement.

### 2.5. Statistical Analysis

The thickness, transmittance, mechanical strength, and surface tension of the PMMA solutions and coagulating baths were tested a minimum of five times. The results of these parameters are presented in the form of mean ± standard deviation. Additionally, ANOVA data analysis was employed in this work to investigate thickness-dependent variations, as shown in Figure S1. For thickness-dependent performance variations, ANOVA revealed a dominant effect (F = 244.94, *p* < 1 × 10^−84^, *R*^2^ = 0.98), with thickness explaining 98% of response differences, supported by a low RMSE (1.91) and C.V. (0.07) indicative of high model precision and data consistency (Figure S1a,b). Concurrently, analysis of optical properties showed that surface tension significantly correlated with transparency (*R*^2^ = 0.91, *p* ≈ 0), accounting for 91% of transparency differences, alongside a robust model reflected in a low C.V. (0.0096) and RMSE (0.85) (Figure S1c,d). For viscosity–transparency relationships, ANOVA demonstrated a highly significant association (F = 244.94, *p* < 1 × 10^−84^, *R*^2^ = 0.98), where viscosity nearly fully explained transparency differences—likely linked to enhanced molecular alignment or suppressed phase separation in high-viscosity systems—with a negligible C.V. (0.07) confirming data consistency (Figure S1e,f). Collectively, these analyses underscore the statistical rigor of our models and the critical roles of thickness, surface tension, and viscosity in governing the studied properties.

## 3. Results and Discussion

**Figure 1:**
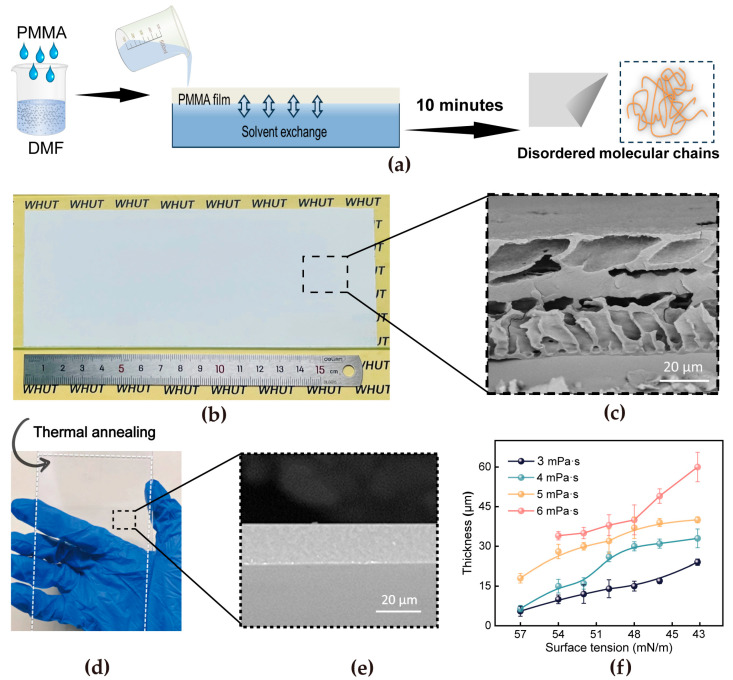
(**a**) Fabrication mechanism of the PMMA film using the superspreading method. (**b**) Optical image of the PMMA film after superspreading and (**c**) SEM image of the PMMA film cross-section. (**d**) Optical image of the PMMA film after thermal annealing and (**e**) SEM image. (**f**) The relationship between the thickness of the PMMA film and the viscosity of the solution and the surface tension of the coagulating bath.

The superspreading method was employed for the continuous preparation of tough and transparent PMMA films with precisely controlled thicknesses (Figure 1a). In this process, the interplay between the viscosity of the polymer solution (η) and the surface tension gradient (Δγ) at the coagulation bath interface governs the film morphology. During the spreading process, the difference in surface tension between the polymer solution and the coagulation bath drives the polymer forward, causing the solution to expand and cover a larger surface area. The viscous dissipation counteracts the fluid motion, thereby stabilizing the expansion dynamics. This ultrafast demixing process, typically completed within a short timeframe of approximately 10 min, kinetically traps polymer chains in non-equilibrium configurations, resulting in a microporous morphology with a disordered molecular arrangement. While the surface appears macroscopically smooth (Figure 1b), the cross-sectional SEM image (Figure 1c) reveals a porous morphology due to incomplete polymer chain packing. To address the issue, a post-treatment protocol involving heating at 200 °C for 10 min was implemented. The thermal annealing process is pivotal in tailoring the microstructure of polymer films, aiming to enhance optical transparency and structural densification. The optical image further reveals a smooth surface topography (Figure 1d), coupled with exceptional transparency and internal densification (Figure 1e). Simultaneously, this technique enables precise thickness engineering of the films through real-time modulation. Systematic investigation reveals that the post-annealing film thickness can be controllably tuned from 5.5 μm to 60 μm through rational modulation of processing parameters (Figure 1f). This thickness tunability arises from the superspreading mechanism, which governs the dynamic equilibrium between solvent extraction kinetics and polymer deposition rates during film formation. Especially under conditions of viscosity (6 mPa·s) and surface tension (57 mN/m), continuous and uniform film formation could not be achieved. The strategy demonstrates significant technological advantages for industrial implementation, achieving rapid thin-film deposition through its inherently replicable self-spreading mechanism. This process requires minimalist yet scalable instrumentation, enabling cost-effective production while effectively lowering operational complexity for large-scale manufacturing systems.

**Figure 2:**
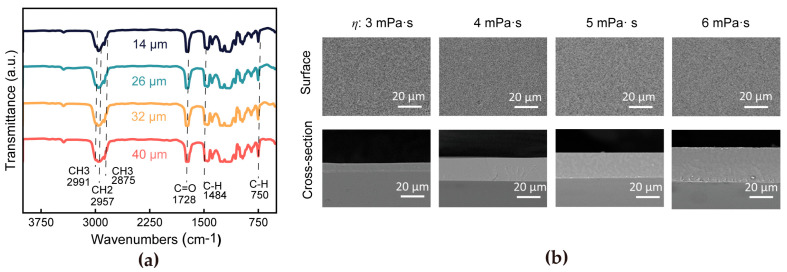
(**a**) FTIR spectra of PMMA films and (**b**) surface morphology and the cross-section of PMMA films with various thicknesses.

Importantly, the superspreading process maintains chemical fidelity regardless of processing conditions, as evidenced by FTIR spectral analysis (Figure 2a). The identical absorption characteristics (ν(C=O) at 1728 cm^−1^, δ(C-H) at 1484 cm^−1^) across all specimens, with less than 2% variation in characteristic peak intensities, confirm that the molecular architecture remains unaffected. This structural preservation highlights a critical advantage of the superspreading mechanism: the ability to modulate physical parameters without compromising molecular integrity, which is essential for functional polymer films requiring strict property consistency. Morphological characterization further validates the method’s superiority. SEM images show that the post-annealed films exhibit uniform thickness, a smooth surface morphology, no defects, and a void-free internal structure (Figure 2b). These microstructural properties directly correlate with the self-leveling nature of the superspreading process, a unique feature that distinguishes it from traditional techniques. The uniformity and defect-free nature of the films are critical for achieving high-quality thin films with consistent mechanical and optical properties, making the superspreading method a reliable approach for industrial applications.

**Figure 3:**
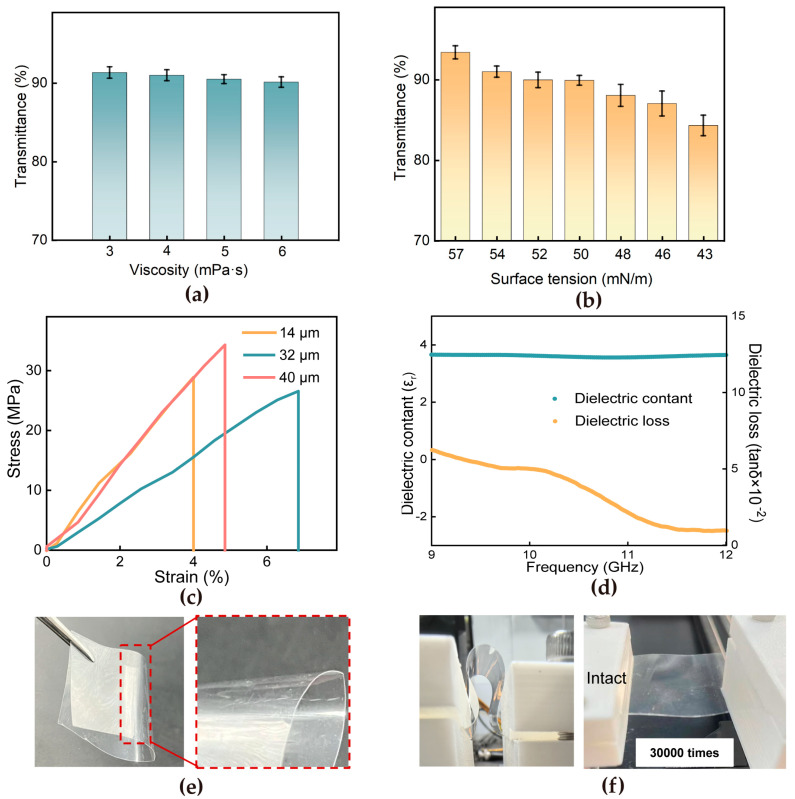
(**a**) The relationship between PMMA film viscosity and transparency at a fixed surface tension of 50 mN/m. (**b**) The correlation between surface tension and transparency at a fixed viscosity of 5 mPa·s. (**c**) Comparison of stress–strain curves of PMMA film across different thicknesses. (**d**) Dielectric constant and dielectric loss. (**e**) Static bending behavior of the PMMA film. (**f**) Dynamic fatigue bending test of PMMA film.

The quantitative UV-Vis spectroscopic analysis demonstrates a systematic correlation between the optical transmittance and rheological parameters of the films. As shown in Figure 3a, the results indicate that an increase in viscosity leads to a slight decrease in film transparency, while Figure 3b reveals that a decrease in surface tension causes a comparable reduction in transparency. Notwithstanding these parameter-dependent variations, all annealed films consistently maintain transmittance values exceeding 85% across the visible spectrum (400–800 nm), satisfying the stringent requirements for optoelectronic applications [35]. Comprehensive tensile testing across different thickness gradients (Figure 3c) shows that the 32 μm film is the best choice, as it has excellent mechanical integrity without affecting its optical performance. Both the 14 μm and 40 μm films exhibited rapid stress decline after reaching their peak values, indicating limited energy dissipation capacity and susceptibility to brittle fracture. On the contrary, the 32 μm film demonstrated a significantly flatter post-yield stress plateau, achieving higher fracture energy compared to the thinner and thicker counterparts. When the thickness of the PMMA film is insufficient, it becomes more fragile or susceptible to plastic deformation. Conversely, when the PMMA film is excessively thick, the material’s stress continues to increase as it undergoes further deformation [36]. This pronounced difference in behavior is the very reason for choosing the 32 μm film. It strikes an ideal balance, avoiding the excessive fragility of the thinner film and the overly rigid and brittle nature of the thicker one. The 32 μm film’s ability to maintain a stable post—yield stress plateau and dissipate energy effectively makes it a superior choice for applications where both strength and toughness are critical. This thickness selection was further validated via dielectric characterization (Figure 3d), which reveals exceptional stability with a dielectric constant of 3.6 ± 0.05 from 9 to12 GHz. The dielectric loss tangent exhibits a favorable downward trend across the measured frequency range. Reduced film thickness enhanced molecular chain packing density, which imposes spatial constraints on dipole mobility. This confinement prolongs the relaxation time (τ) of dipole reorientation, leading to diminished polarization hysteresis and reduced energy dissipation [37].

As illustrated in Figure 3e, the PMMA film sustains complete structural integrity at 180° bending without fracture initiation. Moreover, bending fatigue tests were conducted to assess the durability of both films under repeated bending (Figure 3f). At a fixed curvature of 106 m^−1^, the superspreading-prepared film exhibited no visible surface creases after 30,000 bending cycles, underscoring its superior fatigue resistance. These synergistic characteristics—combining optical transparency, mechanical robustness, and dielectric reliability—establish the PMMA film as a promising candidate for next-generation flexible transparent antenna. The mechanical superiority of the superspreading-prepared PMMA film is further validated by quantifying Young’s modulus (0.38 GPa) and fracture toughness (9 × 10^5^ J/m^3^) from the stress–strain curve, which provide a quantitative basis for interpreting bending fatigue resistance. The high elastic modulus suppresses local plastic deformation during cyclic bending by maintaining structural rigidity, as evidenced by the linear stress–strain relationship in the elastic regime. Concurrently, the enhanced fracture toughness enables efficient crack blunting and deflection mechanisms, dissipating strain energy and preventing catastrophic failure.

**Figure 4:**
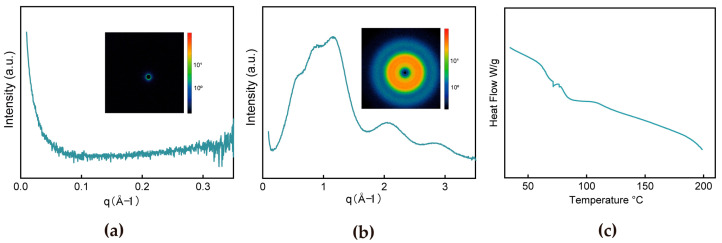
(**a**) SAXS and 2D-SAXS diffraction ring and (**b**) WAXD and 2D-WAXD diffraction ring. (**c**) The DSC curve from 0 to 200 °C.

The multifunctional superiority of superspreading-derived PMMA films—including simultaneous optical transparency (85–95%), mechanical properties, and dielectric stability (ε_r_ = 3.6 ± 0.05)—is fundamentally rooted in their hierarchically disordered molecular architecture, as demonstrated by X-ray scattering. Small-angle X-ray scattering (SAXS) profiles exhibited a featureless intensity decay (Figure 4a) with azimuthally isotropic scattering halos, confirming the absence of nanoscale crystalline domains or phase-segregated structures.

Complementarily, wide-angle X-ray scattering (WAXD) (Figure 4b) displays continuous concentric rings rather than discrete crystalline reflections, corroborating atomic-scale amorphousness—a lack of periodic lattice arrangements. Several of the peaks observed in the plots are due to the crystalline structure of polymers [38,39,40], with bumps indicating the presence of very small microcrystals [41]. The absence of sharp diffraction peaks confirms random spatial distribution of scattering entities, a hallmark of disordered polymer networks. The DSC results (Figure 4c) confirm the amorphous nature of PMMA, as evidenced by the absence of an exothermic crystallization peak in the high-temperature region, which eliminates brittle crystalline defects and enhances material toughness. A distinct step-like drop in heat flow, corresponding to the glass transition temperature (Tg ≈ 70 °C), indicates the onset of coordinated molecular chain motion above this temperature, endowing the material with superior ductility in the high-elastic state. The broadened heat flow variation near Tg suggests localized density fluctuations within the amorphous network, yet the overall homogeneous and disordered structure minimizes dielectric defects, thereby optimizing dielectric strength. This structural homogeneity is attributed to rapid processing techniques that suppress molecular ordering, forming a dense amorphous network. The intrinsic molecular disorder within superspreading-derived films directly reconciles traditionally conflicting material requirements. Disordered chain arrangements simultaneously enhance mechanical properties through energy-absorbing while maintaining transparency via suppressed light scattering. Furthermore, this disordered structure ensures dielectric stability through a uniform polarization response (facilitated by reduced interchain coupling) [42]. while suppressing crystalline-induced anisotropy via atomic-to-nanoscale randomness.

A systematic comparison of conventional PMMA fabrication methods (summarized in Table 1) underscores the superior performance of the superspreading technique in critical parameters for ultra-wideband flexible transparent antennas. The comparison reveals that the superspreading technique offers distinct advantages for preparing PMMA films tailored to ultra-wideband flexible transparent antennas. Specifically, the superspreading technique achieves thickness tunability (1–100 μm) for PMMA films, effectively bridging the gap between ultrathin spin-coated layers (0.1–10 μm) and thick solution-cast/hot-pressed sheets (100–1000 μm). Simultaneously, the films maintain high optical transparency (85–95%), comparable to spin-coating (90–95%), while surpassing other thick-film methods. This high transparency is a prerequisite for integrating antennas into systems transparent to visible light. The technique’s most striking advancement lies in mechanical robustness. Films fabricated via superspreading exhibit exceptional fracture toughness (9 × 10^5^ J/m^3^), nearly doubling the values of other methods (~5 × 10^5^ J/m^3^). This toughness value was calculated by integrating the area under the stress–strain curve of the 32-μm sample, with the integration range spanning from the origin to the breaking point (where the strain reaches 6.85%). This exceptional toughness is directly linked to molecular-level energy dissipation mechanisms. Specifically, the disordered molecular arrangement plays a significant role in enhancing the material’s toughness. The increase in disorder hinders the sliding of molecules or atoms, thereby dispersing external forces more effectively [43]. This reduces stress concentration, improves energy absorption under stress, and ultimately results in enhanced toughness. These findings substantiate the benefits of molecular structural disorder in enhancing the mechanical properties of the material.

**Figure 5:**
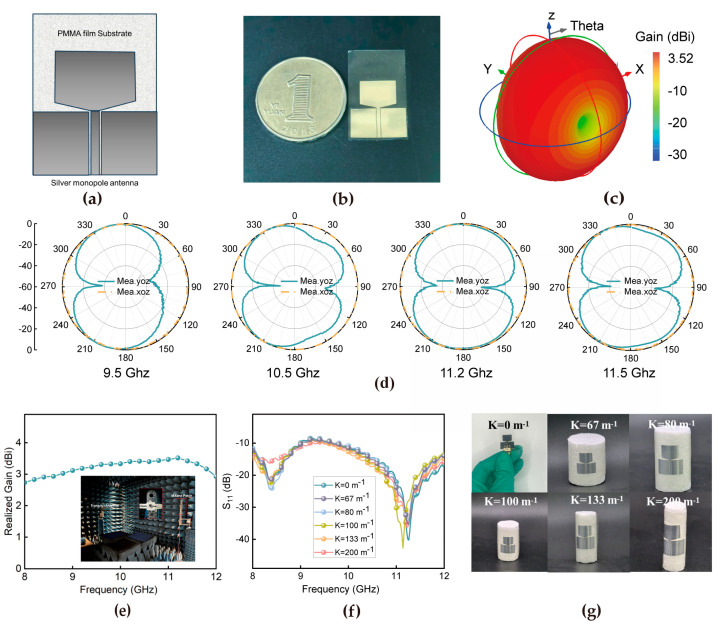
(**a**) Structure design of the antenna. (**b**) Physical drawing of the antenna. (**c**) The 3D directional map at 11.2 GHz. (**d**) Measured radiation patterns for the proposed antenna at 9.5 GHz, 10.5 GHz, 11.2 GHz, and 11.8 GHz. (**e**) Measured gains versus frequency. (**f**) Digital photographs at different bending curvatures. (**g**) Measured reflection coefficients of the antenna at different bending curvatures.

To evaluate the practical application prospects, we designed a coplanar waveguide monopole antenna operating in the 8–12 GHz band using optimized PMMA substrates based on the measured dielectric constant and dielectric loss. The antenna design is illustrated in Figure 5a, while the physical drawing of the antenna is shown in Figure 5b. The transparent portion represents the PMMA film substrate, and the silver portion denotes the conductive layer. The antenna’s conductive layer is thermally evaporated using 99.99% pure silver, ensuring high conductivity. The deposition process is carried out under precise conditions, with a base vacuum of ≤5 × 10^−6^ Torr and a deposition rate of 2 Å/s. The antenna is vapor-plated to guarantee compatibility and consistent performance with the thin film material. While this study primarily focuses on optimizing the antenna performance, the prototype fabrication concentrated on non-transparent versions. However, our permittivity-matching design (ε_r_ = 3.6 ± 0.05 vs. air ε_r_ = 1) theoretically enables excellent optical transparency without sacrificing RF performance [51].

As demonstrated in prior work, this design principle has been successfully validated in transparent antenna implementations using identical dielectric matching criteria, achieving > 80% visible-light transmittance. Our results here provide foundational insights into material parameter optimization, which directly supports the scalability of this approach for future transparent antenna development. The 3D radiation pattern at 11.2 GHz explicitly demonstrates stable azimuthal symmetry (Figure 5c). The omnidirectional radiation characteristics of the monopole antenna across four selected frequency points are systematically illustrated in the directional radiation patterns (Figure 5d). This exceptional radiation performance originates from the superior material properties, particularly the remarkable dielectric stability of the engineered composite film. These optimized material characteristics facilitate efficient electromagnetic energy propagation while maintaining uniform radiation distribution, thereby ensuring consistent omnidirectional radiation patterns throughout the operational frequency band. As evidenced by the gain-bandwidth analysis (Figure 5e), the antenna achieves a peak gain of 3.5 dBi at 11.2 GHz, indicating significantly enhanced radiation efficiency at this specific frequency. Notably, the sustained gain level within 2–4 dBi satisfies the essential requirements for small-to-medium-scale communication devices [52], confirming its potential for ultra-wideband systems. The curvature-dependent S_11_ parameter analysis reveals outstanding impedance matching characteristics (Figure 5f), with reflection coefficients maintaining below −10 dB throughout the 8–12 GHz range under various bending conditions. To characterize the antenna performance comprehensively, we first conducted detailed electromagnetic simulations in CST Studio Suite using its Time-domain Solver, focusing on a coplanar waveguide design integrated with flexible PMMA films. To mitigate the narrow bandwidth issue typical of microstrip feeding, a 50 Ω coplanar waveguide (CPW) feeding method was adopted. Meanwhile, we conducted simulation calculations under different curvatures, as shown in Figure S2. The results indicate that the mechanical stress induced by bending also has a negligible impact on the gain and radiation efficiency, confirming that the PMMA-based antenna maintains stable electromagnetic performance even under flexible deformation. This analysis mainly aims to evaluate the stability of the material’s radio—frequency performance under mechanical stress when used in flexible antennas. Of particular significance is the minimal reflection coefficient (−40.2 dB) observed at 11.2 GHz (Figure 5g), which corresponds to exceptionally low reflection losses. Remarkably, the S_11_ parameters exhibit minimal variation (±0.8 dB) with curvature changes, confirming excellent mechanical stability that is crucial for the integration of flexible electronics.

The calculated relative bandwidth (f_foc_), determined by f_foc_ = 2(f_H_ − f_L_)/(f_H_ + f_L_) × 100% = 40%, surpasses the 25% threshold for ultra-wideband (UWB) classification. This broadband capability, enabled by the material’s frequency-stable dielectric properties, supports high-speed data transmission in UWB systems. The comparative analysis presented in Table 2 demonstrates distinct performance characteristics between this flexible coplanar waveguide monopole antenna design and recently reported counterparts. As evidenced by the data, prior studies have primarily focused on optimizing conventional antenna performance metrics, including gain (2.98–3 dBi) and bandwidth (20.2–36.44%), with limited consideration given to material flexibility. In contrast, this study highlights the pivotal importance of material flexibility in antenna design through the implementation of an ultrathin substrate (0.032 mm thickness) and a high-tolerance bending material architecture. The material-centric flexibility strategy not only ensures exceptional mechanical durability under deformation but also resolves critical reliability challenges associated with repeated bending cycles in flexible electronics. The proposed antenna, with its innovative material-flexible design and competitive operational bandwidth (40%) across the 8–12 GHz frequency range, offers enhanced compatibility for integration into next-generation conformal electronic systems. A comprehensive evaluation confirms that the antenna satisfies all critical performance metrics for modern communication applications. Its enhanced flexibility and operational stability are directly attributable to the disordered molecular chains of the material.

## 4. Conclusions

In conclusion, this study establishes a superspreading fabrication strategy as an effective approach for precisely modulating molecular disorder in PMMA films, thereby achieving significant enhancements in both mechanical and electrical performance. Through this advanced processing method, the engineered films exhibit at least 50% enhancement in toughness, increasing from 4–6 × 10^5^ J/m^3^ to 9.0 × 10^5^ J/m^3^. Notably, fatigue resistance is markedly enhanced, with the films sustaining over 30,000 bending cycles without failure. These breakthrough performance metrics position the superspreading approach as a transformative methodology for developing high-reliability polymer substrates in flexible electronics. Significantly, the synergistic control of molecular orientation through superspreading simultaneously optimizes dielectric stability, enabling successful implementation in ultra-wideband coplanar waveguide monopole antenna design. The prototype device demonstrates outstanding electromagnetic characteristics, including stable omnidirectional radiation patterns (gain variation < 1 dBi) and broadband impedance matching (S_11_ < −10 dB over 8–12 GHz), thereby fulfilling critical requirements for next-generation ultra-wideband (UWB) systems. This methodology fundamentally advances polymer processing technology by establishing quantitative structure-property relationships between molecular disorder and multifunctional performance.

## Figures and Tables

**Table 1:** Performance comparison of PMMA films fabricated through conventional methods.

Method/ Parameter	Thickness (μm)	Tensile Strength MPa	Young’s Modulus (GPa)	Elongation at Break (%)	Toughness (×10^5^ J/m^3^)	Transmittance (%)
Solution-Casting [18,19,44,45,46]	100–1000	30	2	2	5	85–90
Hot-Pressing [47,48]	100–1000	23.4	1.67	1.8	6	80–90
Spin-Coating [20,21,22]	0.1–10	50	2	5	4	90–95
NIPS [49,50]	1–100	60	0.5	4	5	90–95
Superspreading	1–100	26.6	0.38	6.85	9	85–95

**Table 2:** Comparison of recently reported flexible coplanar waveguide monopole antennas.

Ref	Gain (dBi)	Bandwidth (%)	Size (mm × mm × mm)	Substrate Dielectric Constant	Bending Radius (m^−1^)	Frequency (GHz)
This study	2–4	40	0.032 × 15 × 25	3.6	200	8–12
[53]	2.98	28.33	40.6 × 50 × 0.075	3.5	25	2.28
[54]	1.69	30	30.4 × 38 × 0.07	3.5	100	3.1–10.6
[55]	3	36.44	40.6 × 50 × 0.075	3.5	16.67	2.4
[56]	1.5–3	20.2	25 × 20 × 1.6	4.4	\	2.4–5

## Data Availability

The original contributions presented in this study are included in the article. Further inquiries can be directed to the corresponding authors.

## Supplementary Materials

The following supporting information can be downloaded at: https://www.mdpi.com/article/10.3390/ma18102183/s1, Figure S1: Analysis of variance (ANOVA); Figure S2: Antenna simulation.
