# Supplementary material for: Superspreading-Based Fabrication of Poly(methyl methacrylate) Films with High Toughness for Ultra-Wideband Flexible Transparent Antenna
Source: Materials (Basel). 2025 May 9;18(10):2183. doi: 10.3390/ma18102183 (PMC12112956; doi:10.3390/ma18102183)
Supplement: Supplementary file 1 [file materials-18-02183-s001.zip › materials-3571745-supplementary.pdf]

Superspreading-based Fabrication of Poly(methyl methacrylate) Films with High Toughness for Ultra-Wideband Flexible Transparent Antenna

Supplementary Figure S1-S2

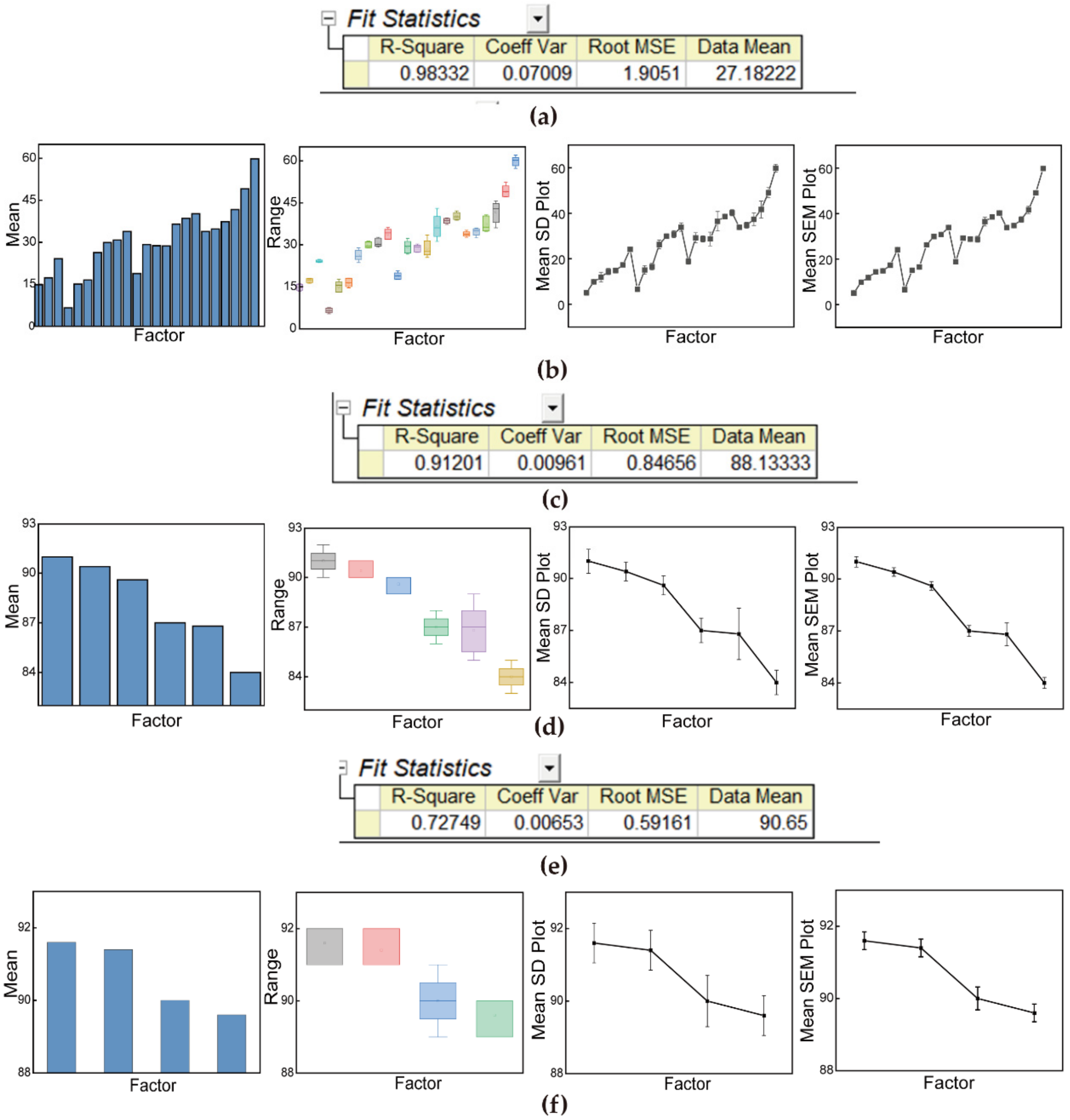

Fit Statistics

R-Square	Coeff Var	Root MSE	Data Mean
0.98332	0.07009	1.9051	27.18222

Fit Statistics

R-Square	Coeff Var	Root MSE	Data Mean
0.91201	0.00961	0.84656	88.13333

Fit Statistics

R-Square	Coeff Var	Root MSE	Data Mean
0.72749	0.00653	0.59161	90.65

Figure S1. (a) Fit statistics and (b) mean value distribution: viscosity and surface tension as functions of thickness, (c) Fit statistics and (d) mean value distribution: transparency as a function of thickness, (e) Fit statistics and (f) mean value distribution: transparency as a function of viscosity.

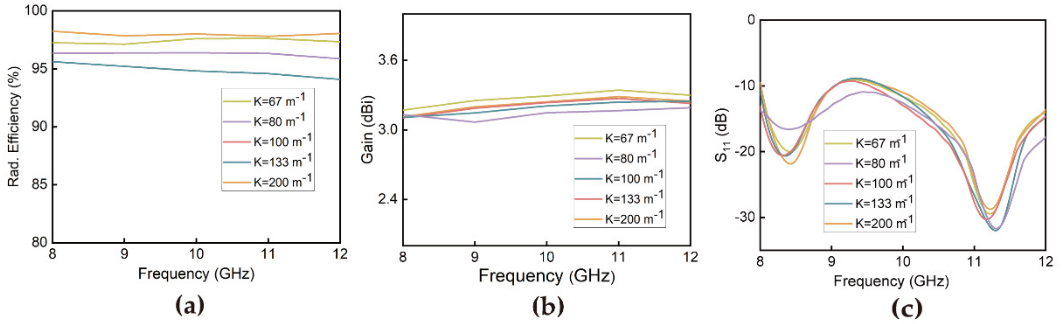

Figure S2. (a) Simulation radiation efficiency, (b) gain, and (c) S11 reflection coefficient of the antenna at different bending curvatures.
